# Effect of calcium hypochlorite and sodium hypochlorite on postoperative pain in necrotic pulps: A randomized clinical trial

**DOI:** 10.4317/jced.62797

**Published:** 2025-10-17

**Authors:** Luciana Oliveira Leal, Matheus Albino Souza, João Paulo De Carli, Pedro Henrique Corazza, Doglas Cecchin, Yuri Dal Bello

**Affiliations:** 1 Universidade de Passo Fundo, Campus I, Faculdade de Odontologia, BR 285, Km 171, Bairro São José, Caixa Postal 611, 99052-900, Passo Fundo, RS, Brazil

## Abstract

**Background:**

This randomized clinical trial evaluated postoperative pain and analgesic intake in patients with pulp necrosis and asymptomatic chronic apical periodontitis.

**Material and Methods:**

Sixty-three patients were randomly assigned to two groups receiving root canal irrigation with either 2.5% calcium hypochlorite (Ca (OCl)2) or 2.5% sodium hypochlorite (NaOCl). Root canal preparation was performed using rotary instruments, and all canals were filled in a single visit. Postoperative pain was assessed using a 0-10 numerical rating scale at 24, 48, and 72 hours, and one-week post-treatment. Analgesic intake was also recorded. Statistical analysis was performed using Fisher's exact test.

**Results:**

No significant differences in pain levels were observed between groups at 24 hours (p = 0.601) or other time points. Most patients (95.2%) reported no pain, 3.1% mild pain, and 1.5% severe pain. Analgesic intake was reported by only one patient.

**Conclusions:**

Calcium hypochlorite is a promising irrigant for endodontic treatment, showing comparable postoperative pain outcomes to sodium hypochlorite.

## Introduction

During root canal preparation with hand, rotary, or reciprocation instruments, dentin chips, organic tissue, and microorganisms can accumulate in the anatomical complexities of the root canal system, such as isthmuses, irregularities, and ramifications (1). In this context, endodontic solutions plays a fundamental role in reducing infected tissues, as well as in areas that remain untouched by the instruments (2). Sodium hypochlorite (NaOCl) is the most used irrigant for root canal irrigation (3). It is used due to its antibacterial and tissue-dissolution properties. However, these beneficial effects are often associated with undesirable side effects, such as the breakdown of the collagen matrix within the mineralized dentin structure (4) and cytotoxicity to periapical tissues (5). Consequently, biosafety concerns related to NaOCl, particularly when used at high concentrations or for prolonged periods during root canal therapy, underscore the need for the development of novel irrigants specifically designed to enhance canal debridement. Calcium hypochlorite (Ca(OCl)2) is a halogenated compound commonly used for industrial sterilization, bleaching, and water purification (6). This alkaline white powder, soluble in water, has demonstrated strong antimicrobial activity against endodontic pathogens, including Enterococcus faecalis, which is known for its resistance and role in endodontic infections (7). In addition to its effective tissue dissolution capacity, calcium hypochlorite exhibits advantages such as a higher available chlorine content and a broader spectrum of microbial action (8). Unlike NaOCl, calcium hypochlorite does not significantly affect the mechanical properties of root dentin, preserving its structural integrity (9). Furthermore, Blattes et al. (10) reported that Ca (OCl)2 showed favorable outcomes in terms of cell migration, viability, and inflammatory response. Additionally, Coaguila-Llerena et al. (11) found Ca (OCl)2 to be less cytotoxic than NaOCl. These combined antimicrobial efficacy and biocompatibility findings support the potential use of Ca(OCl)2 as an irrigating solution for endodontic procedures. However, further clinical studies are necessary to evaluate its clinical performance in endodontics. Postoperative pain (PP) is an unpleasant sensation that occurs following endodontic procedures, affecting between 3% and 58% of patients, regardless of the pulp and periradicular conditions (12). The development of PP after root canal treatment is typically attributed to an acute inflammatory response in the periradicular tissues.(12 , 13). One intraoperative factor that may contribute to PP is the irrigation protocol used during root canal preparation (14 , 15). However, no randomized clinical trial has evaluated PP in roots instrumented with calcium hypochlorite (Ca (OCl)2) as an irrigant. Therefore, the aim of this randomized clinical trial study was to investigate postoperative pain and analgesic intake after endodontic treatment using Ca (OCl)2 and NaOCl solution in asymptomatic nonvital single-rooted teeth with chronic apical periodontitis and pulp necrosis. The null hypothesis tested is that there is no statistically significant difference in pain levels and analgesic intake between the tested solutions.

## Material and Methods

**Figure 1:**
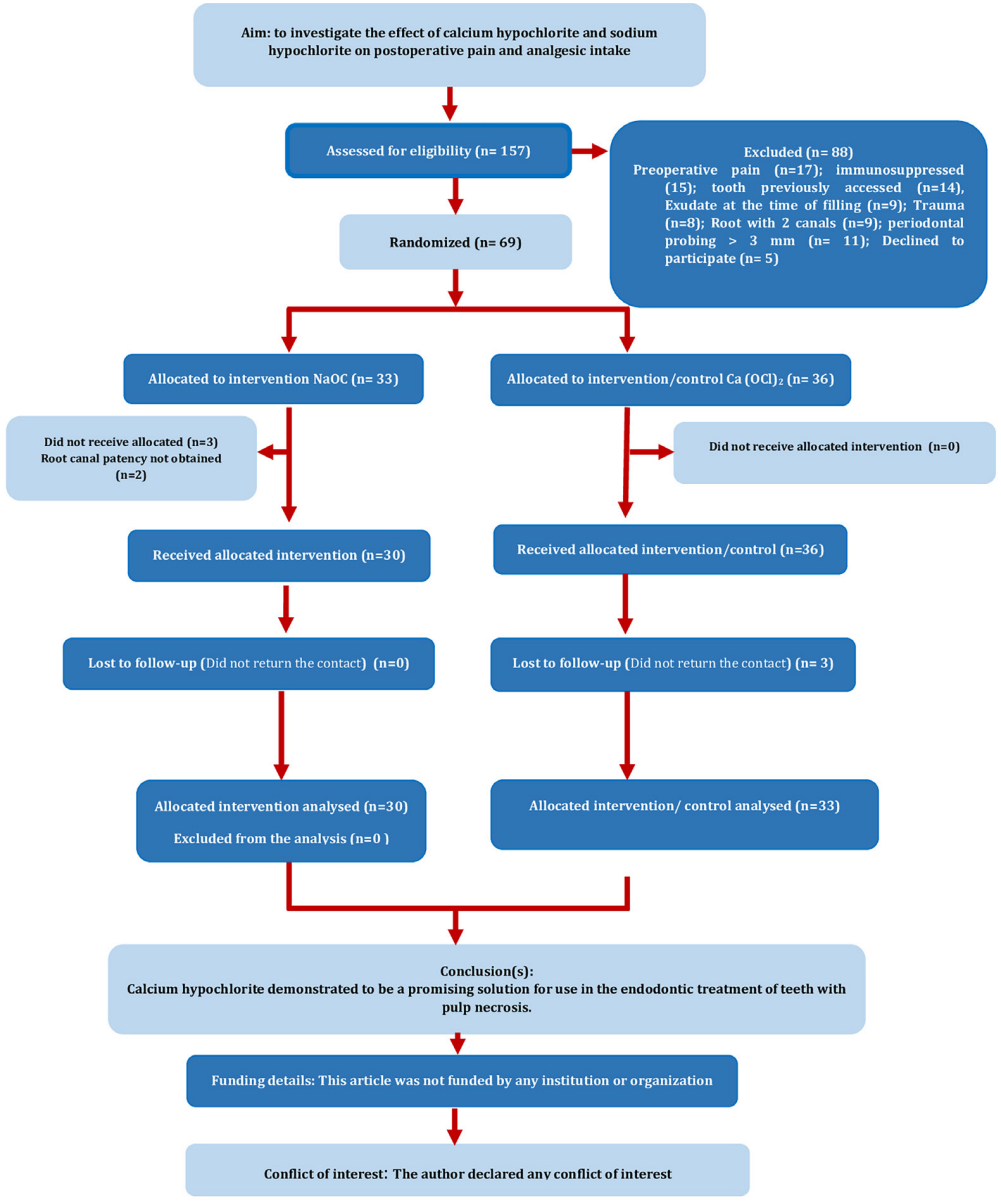
PRIRATE flow chart.

This randomized clinical trial has been written according to the Randomized Trials in Endodontics (PRIRATE) 2020 guidelines (16). This study was approved by the Research Ethics Committee (protocol no. 2.628.763) and registered in the Brazilian Registry of Clinical Trials (Registro Brasileiro de Ensaios Clínicos, http://www.ensaiosclinicos.gov.br/, database no. RBR-4t2hqqt). - Sample size calculation A pilot study was conducted to determine the number of required patients for two groups. A protocol was followed similar to the main study as mentioned below. Twenty patients were randomly divided into two groups. According to the data obtained from the pilot study, the minimum number of patients required for each group was determined to be twenty-nine (two tailed; effect size 0.750; 80% power; alfa error 0.05). Considering missing patients during the follow-up, sexty-nine patients were decided to enrol in the study. - Eligibility criteria Eligible patients for inclusion in this study were systemically healthy subjects between the age of 18 and 80 years with single rooted teeth presenting nonvital pulp and with radiographic evidence of apical periodontitis; asymptomatic patients. For diagnosis, a radiograph was taken and sensitivity testing was carried out with a cold spray (EndoTest, Coltene Whaledent, Langenau, Germany) and hot test with heated gutta-percha stick. Patients were excluded if they were pregnant or lactating; had a history of sensitivity or adverse reactions to any of the medications or materials used in this study; retreatment cases; or had severely curved root canals. Patients who took a pre operative premedication that could alter pain perception (e.g. analgesics) within at least 12 h before treatment were also excluded. Teeth with no apical patency during the treatment; calcified root, periodontal probing &gt; 3 mm, any trace of exudate, or uncompleted root development. Patients with immunosuppression or immunocompromise were also removed. - Solution preparation Calcium hypochlorite powder (Ca (OCl)2) with 65% purity ((Farmaquímica S.A. Produtos Químicos) was weighed on a precision balance. A total of 3.85 g of the powder was dissolved in 100 mL of distilled water to obtain a 2.5% solution (17). Sodium hypochlorite was obtained from commercial formulation with 2,5% concentration (Asfer Indústria Química, SP, Brazil). - Randomization Informed consent was obtained from all participants. Among 157 patients registered between June 2018 and April 2023, sixty-nine were considered eligible based on the inclusion and exclusion criteria. Randomization was performed using an equal allocation ratio, with assignment codes concealed in envelopes. These codes were assigned sequentially to the eligible patients. A clinical investigator, in accordance with the randomization code, prepared and administered the assigned solutions during the root canal procedure. It was ensured that neither the patient nor the operator was aware of the irrigant used, (Fig. 1). - Clinical procedure All clinical procedures were performed by a single practitioner with 15 years of clinical experience in endodontic treatments. The patients received local anesthesia of 2% lidocaine with 1:80,000 epinephrine prior to commencement of the root canal treatment. After rubber dam isolation, the access cavity was prepared, the pulp chamber was flushed with 5 mL the respective solution under study. PathFile (#0.16) rotary files were selected for glide path preparation, and the working length was established with an electronic apex locator (Propex, Maillefer, Ballaigues, Suíça) with size 15 K-files. The file was placed in the canal until the apex locator display indicated "APEX". The silicone stop was calibrated to the reference point, and the length was recorded as the working length. Root canal preparation was performed using the ProDesign Logic rotary system (Easy Equipamentos Odontológicos, Belo Horizonte, MG, Brazil). The #10 Flex-R file was inserted up to the apical foramen followed by a 25/.01 file to achieve patency. The files 30/.01, 30/.05 and 35/.01 were then introduced using an 'in-and-out' motion up to the working length. At each instrument change during the procedure, a #10 Flex-R file was used to confirm patency, and the root canals were irrigated with 3 mL of 2.5% Ca (OCl)2 or 2.5% NaOCl with a 30-gauge needle (Ultradent Products Inc, South Jordan, UT). The syringe with the test solution was handed to the operator by an assistant. Furthermore, the operator was blinded to the solution. The needle was inserted up to 3 mm short of the working length, using a rubber stop as a guide, and the root canal was irrigated, whilst the needle was moved up and down. The smear layer removal protocol involved the use of 5 mL of the EDTA, which was delivered along the entire length of the root canal. The solution was agitated using passive ultrasonic irrigation (PUI) with an E1 tip (Irrisonic, Helse Ultrasonic, Ribeirão Preto, SP, Brazil) attached to a Satelec ultrasonic device (Acteon Group, Merignac, France) at 20% intensity. The final irrigating solution was activated in three cycles of 20 s, with the solution renewed after each cycle (18). The EDTA was removed from the canal by 3 mL saline irrigation. After root canal preparation and irrigation procedures, the root canals were dried with paper points and then filled with gutta-percha and AHPlus (Dentsply, DeTrey, Konstanz, Germany) root canal sealer using the cold lateral condensation technique. After obturation, the access cavity was restored permanently with composite resin and an immediate postoperative radiograph was taken to observe the quality of treatment. At the end of the session, each patient received a rescue bag of 10 Ibuprofen (400 mg) in case they had pain with instructions for on-demand use. - Pain evaluation Before the administration of local anesthesia, all patients were asked about pain in the teeth to be treated. To continue in the study, patients had to report a complete absence of pain; those with any level of pain were excluded. After endodontic treatment, the volunteers were asked by a blind examiner to record their pain perception on a numerical rating scale (NRS/ 0-10). Pain levels were classified as none (0), mild (1-3), moderate (4-7), or severe (8-10). Pain perception and analgesic intake were recorded at intervals of 24, 48, and 72 hours, as well as 7 days post-intervention. Patients were contacted by phone and sent a photo of the NRS scale with their recorded pain level. Additionally, patients were informed that they could request an office appointment if the pain was too intense; in such cases, the event was labeled as a flare-up. - Statistical analysis Baseline demographic, clinical features and pain level of patients were analyzed by non-parametric Ficher Exact test using IBM SPSS Statistics for Windows, version 25.0 software (IBM Corp, released 2017: IBM Corp), and the significance level of all analyses was 5%.

## Results

Sixty-nine participants were included in the study. Six patients were excluded because they failed to show up for postoperative reviews after endodontic treatment (n=3), no root canal patency obtained (n=2) and fracture of endodontic file (n=1) (Fig. 1).

1

Of the sixty-three patients, 38 (60.3%) were women and 25 (39.7%) were men. The average age was 43.5 years (19-68). Thirty-six teeth (57.2%) of the treated teeth were in maxilla and 27 (42.8%) were in mandible. Forty-five teeth (71.4%) were anterior teeth and eighty (28.6%) were in premolar (Table 1).

Table 1

The overall incidence of postoperative pain after RCT during the follow-up period of 24, 48, 72h and 7 days was assessed according to patient's record in the NRS scale. For the application of Fisher's Exact Test (p &lt; .05), pain levels were grouped, as the occurrence of zero frequencies precluded the use of the chi-square test. Data from the 72-hour and 7-day time points did not meet the criteria for statistical analysis. Fisher's Exact Test indicated no statistically significant difference for the irrigants (p = 0.6011 at 24 hours and p = 1.000 at 48 hours) at the 24-hour and 48-hour time points. Sixty patients (95.2%) developed no pain, 2 patients (3.1%) had mild pain, and 1 patient (1.5%) had severe pain. The patient with severe pain shows swelling after 24h, was classified as flare-up and retreatment was the option determined by patient and dentist (Table 2).

Table 2

There was no statistically significant difference in the presence of post-operative pain between the maxilla and mandible (p&gt;0.05). Additionally, no significant differences were found among the central incisor, lateral incisor, canine, and premolar single-rooted tooth groups (p&gt;0.05), nor in the average number of analgesic tablets used between these groups (p&gt;0.05). Analgesic intake was only reported by the patient who experienced a flare-up, using three pills within a 24-hour period.

## Discussion

Postoperative pain (PP) following root canal treatment can be challenging for the dentist and uncomfortable for the patient. The aim of this study was to evaluate PP and analgesic consumption in cases where the root canals were irrigated with 2.5% calcium hypochlorite (Ca (OCl)2) or 2.5% sodium hypochlorite (NaOCl). The results revealed no significant difference in postoperative pain or analgesic intake at any of the time points assessed between the two irrigants. Consequently, the null hypothesis was accepted. Mostafa et al. (14) reported a relatively high incidence of postoperative pain following endodontic irrigation with different concentrations of sodium hypochlorite (1.3% vs. 5.25%) in mandibular molars with necrotic pulps. Their sample included both symptomatic and asymptomatic teeth, which may have contributed to the higher pain levels observed. Additionally, the treatments were performed by nine different operators, potentially introducing variability in clinical procedures and pain outcomes. Preoperative pain is a well-established predictor of postoperative pain (19). To minimize bias, all participants in this study were required to report no pain at the beginning of the study, and all treatments were performed by the same operator to ensure consistency in the clinical procedures. Others factors such as age, sex, and tooth position have been identified as potential influences on postoperative pain (20). Previous studies have highlighted associations between increased pain and variables like older age, mandibular teeth, and sex, with women often experiencing more pain (21). In the present study, no significant associations were found between factors such as age, sex, or dental arch (p &gt; 0.05). These results differ from those reported by Arias et al., (22) where the authors reported that the probability of moderate or severe pain is higher in mandibular teeth and by Pamboo et al., (21) who observed that women were more frequently affected. Flare-ups are defined as the onset or continuation of pain and/or swelling after endodontic treatment (23). In the present study, all patients presented periapical lesions without preoperative pain, and only one participant (1.5%) experienced a flare-up after treatment with sodium hypochlorite. This low incidence may be attributed to the operator's experience (15 years), who performed all treatments following a standardized protocol and controlled kinematics to minimize debris remnants or extrusion, thus reducing the likelihood of postoperative pain. In the present study, all treatments were performed in a single visit. This approach was adopted to exclude the variable 'intracanal medication' from the analysis. It is reported that calcium hydroxide (CH) has potential to avoid the irrigant effect on postoperative pain (24). Additionally, no significant difference in postoperative pain was reported between single and multiple-visit treatments (23). Different scales have been used to evaluate postoperative pain. The questionnaires must be understood by patients and lend to straightforward interpretation (25). In this study, the numerical rating scale (NRS) was used, as it is widely recognized for pain evaluation due to its reliability, cross-cultural adaptability, and superior sensitivity compared to other methods (26). Additionally, the NRS allows patients to independently report their pain levels, minimizing potential bias introduced by researchers or operators (27). In the present investigation the overall incidence of postoperative pain in root canal treatment was 4.7%. Although low, this value is within the average reported for postoperative pain after endodontic treatment 3-58% (12). Furthermore, no pain was registered at 72 h and 7 days being reported only in the first 48 hours of evaluation. Similar results were reported by Pak and White (13) with significantly decrease in pain levels after 48h. In addition, there was no significant difference in analgesic intake between the groups (p&gt;0.05). Similar results were reported by Silva et al., (28) who found no significant difference in the number of analgesic pills consumed when comparing instrumentation with 5.25% sodium hypochlorite and 2% chlorhexedine. One of the intraoperative factors contributing to postoperative pain is irrigant used. (15) NaOCl is associated with a dose-dependent antimicrobial and tissue-dissolving effect that increases with higher concentrations, however is accompanied by higher cytotoxicity and a greater risk of extrusion periapically (3 , 14). In this study, we opted for a 2.5% concentration of sodium hypochlorite, as it is associated with a lower incidence of postoperative pain (29). Calcium hypochlorite powder with 65% purity was used as the starting material and diluted to prepare a 2.5% solution for clinical application. This approach was selected due to the commercial availability and practicality of the 65% powder formulation for initial clinical research. Although 65% purity calcium hypochlorite is not yet commonly used in endodontics, it provided a feasible means to prepare the irrigant solution at the desired concentration. We acknowledge that formulations with higher purity may present different chemical and clinical characteristics and recommend future studies to explore these alternatives. Calcium hypochlorite is a relatively stable chemical widely used in water purification and industrial sterilization (30) It has a higher amount of available active chlorine and exhibits a lower inflammatory response compared to sodium hypochlorite (8 , 10). Furthermore, it has demonstrated the ability to reduce Enterococcus faecalis in root canals (7). We selected a 2.5% concentration of calcium hypochlorite, as it has shown high antimicrobial efficacy with minimal collagen damage (31). Although calcium hypochlorite has shown promising results as an endodontic irrigant, further studies are essential to fully understand its potential and limitations. The current evidence indicates its antimicrobial properties and effectiveness in tissue dissolution, but there is a need for more controlled clinical trials to evaluate its long-term efficacy, biocompatibility, and safety in various clinical settings. Additionally, future research should explore optimal concentration levels, application techniques, and possible interactions with other endodontic materials. One important limitation of the present study is the low incidence of postoperative pain observed in both groups, particularly after the first 24 hours. While a post-hoc power analysis confirmed adequate statistical power based on the 24-hour outcome (80.2%), the low frequency of events may have reduced the ability to detect small but potentially clinically relevant differences between the irrigants. This increases the risk of a type II error, meaning that a true difference may have gone undetected. Therefore, future clinical trials with larger sample sizes and more sensitive pain assessment strategies such as continuous pain scales are warranted to confirm and expand upon these findings. This study has some limitations that should be acknowledged. First, the low incidence of postoperative pain in the sample, particularly due to the inclusion of only asymptomatic patients, may have reduced the statistical power to detect subtle but clinically relevant differences between the irrigants. Additionally, the relatively small sample size and the short follow-up period may limit the generalizability of the findings. Furthermore, only one operator performed all treatments, which, while reducing procedural variability, may limit external validity. Future studies with larger and more diverse patient populations, longer follow-up times, and multicenter designs involving multiple operators are recommended to confirm and expand upon these findings. Moreover, the investigation of different concentrations and formulations of calcium hypochlorite, as well as more sensitive and quantitative pain assessment tools, would provide a deeper understanding of the clinical performance of this irrigant.

## Conclusions

In conclusion, the incidence of postoperative pain and the need for analgesic medication were similarly low at all postoperative time points for both calcium hypochlorite and sodium hypochlorite. These findings suggest that, at the tested concentrations, both irrigants perform comparably in terms of postoperative comfort. Calcium hypochlorite demonstrated clinical safety and shows promise as an effective irrigant, particularly in cases of pulp necrosis. However, given the low overall incidence of pain in this study, small but clinically relevant differences between irrigants may not have been detected. Therefore, further randomized clinical trials with larger sample sizes and more sensitive pain assessment methods are recommended to confirm these results and to optimize the use of calcium hypochlorite in routine endodontic practice.

## Figures and Tables

**Table 1:** Baseline demographic and clinical features of patients in study groups.

Baseline demographic and clinical features	NaOCl, n (%)(n= 30)	Ca (OCl)2, n (%) (n=33)	Total (n=63)	P value*
Age	41.2 ± 13.4	42.5 ± 12.2		>0.05
GenderFemaleMale	17 (56.7%)13 (43.3%)	17 (48.5%)16 (51.5%)	34 (53.9%)29 (46.1%)	>0.05
Maxillary teethMandibular teeth	19 (52.8%)14 (51.8%)	17 (47.2%)13 (48.2%)	3627	>0.05
Central incisor	8 (53.3%)	7 (46.7%)	15	>0.05
Lateral incisor	10 (45.5%)	12 (54.5%)	22	
Canine	2 (25%)	6 (75%)	8	
PM	8 (44.5%)	10 (55.5%)	18	

* Fisher’s Exact Test (p < .05)

**Table 2:** Postoperative pain level.

	NaOCl (%) (n=30)				Ca (OCl)2 (%) (n=33)			
	Pain level				Pain level			
	None	Mild	Moderate	Severe	None	Mild	Moderate	Severe
24 h	28 (93.3)	1 (3.3)	0	1 (3.3)	32 (97)	1 (3)	0	0
48 h	30 (100)	0	0	0	32 (97)	1 (3)	0	0
72 h	30 (100)	0	0	0	33 (100)	0	0	0
7 days	30 (100)	0	0	0	33 (100)	0	0	0

2

## Data Availability

The datasets used and/or analyzed during the current study are available from the corresponding author.
